# Molecular and Dual-Isotopic Profiling of the Microbial Controls on Nitrogen Leaching in Agricultural Soils under Managed Aquifer Recharge

**DOI:** 10.1021/acs.est.3c01356

**Published:** 2023-07-19

**Authors:** Laibin Huang, Elad Levintal, Christian Bernard Erikson, Adolfo Coyotl, William R. Horwath, Helen E. Dahlke, Jorge L. Mazza Rodrigues

**Affiliations:** † Department of Land, Air, and Water Resources, University of California, Davis, Davis, California 95616, United States; ‡ Environmental Genomics and Systems Biology Division, Lawrence Berkeley National Laboratory, Berkeley, California 94720, United States

**Keywords:** NO_3_^−^ leaching, dual isotopes, metagenome, nitrification, denitrification

## Abstract

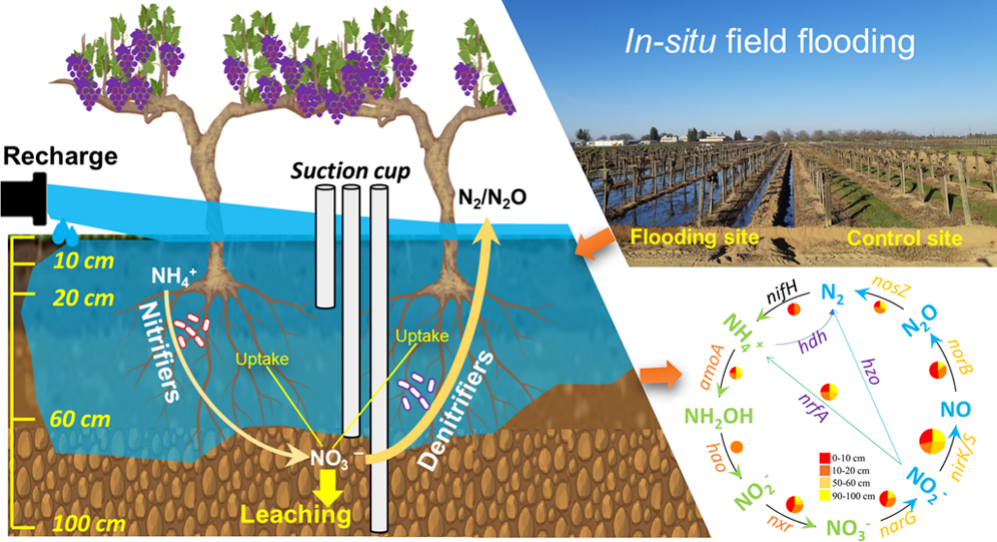

Nitrate (NO_3_^–^) leaching is a serious health and ecological concern in global agroecosystems, particularly those under the application of agricultural-managed aquifer recharge (Ag-MAR); however, there is an absence of information on microbial controls affecting NO_3_^–^ leaching outcomes. We combine natural dual isotopes of NO_3_^–^ (^15^N/^14^N and ^18^O/^16^O) with metagenomics, quantitative polymerase chain reaction (PCR), and a threshold indicator taxa analysis (TITAN) to investigate the activities, taxon profiles, and environmental controls of soil microbiome associated with NO_3_^–^ leaching at different depths from Californian vineyards under Ag-MAR application. The isotopic signatures demonstrated a significant priming effect (*P* < 0.01) of Ag-MAR on denitrification activities in the topsoil (0–10 cm), with a 12–25-fold increase of ^15^N–NO_3_^–^ and ^18^O–NO_3_^–^ after the first 24 h of flooding, followed by a sharp decrease in the enrichment of both isotopes with ∼80% decline in denitrification activities thereafter. In contrast, deeper soils (60–100 cm) showed minimal or no denitrification activities over the course of Ag-MAR application, thus resulting in 10–20-fold of residual NO_3_^–^ being leached. Metagenomic profiling and laboratory microcosm demonstrated that both nitrifying and denitrifying groups, responsible for controlling NO_3_^–^ leaching, decreased in abundance and potential activity rates with soil depth. TITAN suggested that *Nitrosocosmicus* and *Bradyrhizobium*, as the major nitrifier and denitrifier, had the highest and lowest tipping points with regard to the NO_3_^–^ changes (*P* < 0.05), respectively. Overall, our study provides new insight into specific depth limitations of microbial controls on soil NO_3_^–^ leaching in agroecosystems.

## Abstract 2

Conducting groundwater recharge in agricultural systems requires understanding of the biological controls of nitrate leaching and nitrogen cycling processes.

## 1 Introduction

Groundwater serves as a primary freshwater source for over 25% of the world population and ∼40% of the global agricultural ecosystems.^1−5^ However, it has been overexploited within the last century, especially in the arid and semiarid regions due to increasing water demand from growing human and animal populations and climate change.^4,6,7^ Managed aquifer recharge (MAR) has been proposed as an alternative to maintain and secure the quantity and quality of groundwater using surface water sources, which is also referred to as agricultural-MARS or Ag-MAR, where on-farm recharge is implemented in agroecosystems.^3,8−11^ Nevertheless, Ag-MAR application is not free of risks to the environment, and one of its largest drawbacks is nitrate (NO_3_^–^) leaching to the underlying groundwater.^3,4,6,12,13^ It has been reported that 50–60%^14,15^ or even over 137–145%^16^ of the initial residual soil NO_3_^–^ could be leached down to the groundwater, highlighting a profound lack of understanding of microbial controls on N dynamics under Ag-MAR.

Nitrification and denitrification are two major microbial processes controlling NO_3_^–^ transformation under Ag-MAR. Although dissimilatory nitrate reduction to ammonia (DNRA) and anaerobic ammonium oxidation (annamox) are possibly active under flooding conditions, they are minor contributors to NO_3_^–^ removal.^17,18^ Nitrification is currently the only known microbial process that can transform ammonia (NH_3_) to nitrite (NO_2_^–^) and nitrate (NO_3_^–^), thereby greatly contributing to NO_3_^–^ leaching and/or providing substrates for the nitrogen removal processes.^19,20^ Numerous studies on the ecophysiology of nitrifiers have shown that the reduction of nitrification activity using organic fertilizers and nitrification inhibitors can decrease NO_3_^–^ leaching in agricultural ecosystems.^21,22^ However, there is a dearth of information on deep soil nitrifiers that potentially contain vast phylogenetic and metabolic diversity. Particularly, a comprehensive inquiry into their vertical distribution, activities, as well as the niche differentiation is lacking in the subsurface soils,^23^ which thus far impedes our understanding of their response to Ag-MAR applications in the vadose zone of agricultural soils.

In contrast, denitrifiers have received more attention in MAR applications in deep agricultural soils. Gorski et al.^18^ and Beganskas et al.^24^ have both shown that carbon-rich permeable reactive barriers (PRBs) enriched deep soil denitrifiers with increased NO_3_^–^ removal in Ag-MAR events. Likewise, Chen et al.^25^ reported that the denitrification rate was significantly increased as the abundance of *nirK/S* genes and denitrifier populations (e.g., *Pseudomonas* and *Bacillus*) were enriched by the increased organic carbon availability in different soil depths, particularly in the subsurface below 0.5 m. Besides, NO_3_^–^ removal is also reported to be largely affected by the infiltration rate,^17,18,24,26^ the soil texture, and the flooding frequency/duration.^16,27^ However, the high-resolution (e.g., metagenomic profiling) and depth-specific distribution of nitrifiers and denitrifiers, as well as the environmental factors that shape their competition and coexistence, have not been systematically explored in agricultural soils to capture a full understanding of the microbial controls on NO_3_^–^ leaching during Ag-MAR application.

The aim of our study is to (1) investigate the high-resolution and depth-specific distribution of nitrifiers and denitrifiers and the environmental controls on their assembly, as well as (2) quantify nitrification and denitrification activities during NO_3_^–^ leaching in the vadose zone of agricultural soils subjected to Ag-MAR practice. Our hypothesis is that as soil conditions change with depth, there will be depth-specific patterns related to the activities and structures of nitrifiers and denitrifiers. However, short-term Ag-MAR events are expected to only affect the activities of these microorganisms, not their structures. We further predict that NO_3_^–^ leaching will become more prominent by time due to the decreases in the denitrification activity.

## 2 Materials and Methods

### 2.1 Field Experiment and Sampling

*In situ* field flooding (Figure S1) was conducted in two Thompson seedless grape vineyards (*Vitis vinifera* L.) at Kearney Agricultural Research and Extension Center (36.6008°N, 119.5109°W), which is 20 km southeast of the City of Fresno, California. The site has a semiarid, Mediterranean climate, and the soil texture of the two vineyards consists of 58–81% of sand, 4–9% of clay, and 14–32% of silt (Figure S2), classified as a Hesperia series with a deep fine sandy loam (coarse-loamy, mixed, superactive, nonacid, thermic Xeric Torriorthents) for the large vineyard (V1) and Hanford series with a fine sandy loam (coarse-loamy, mixed, superactive, nonacid, thermic Typic Xerorthents) for the small vineyard (V2). We divided each vineyard into six individual subplots, of which three were flooded and three were control plots. V1 was flooded for 4 weeks with an infiltration rate of ∼0.088 ± 0.031 m/day, while V2 was flooded for 2 weeks with an infiltration rate of ∼0.171 ± 0.025 m/day. Groundwater was used as the water source (with 2–3 mg/L of NO_3_^–^-N) and flooding started automatically at 06:00, 14:00, and 22:00 for 2–3 h at each time in March 2020. A total of 200 soil samples (triplicate included) were collected with a core sampler (diameter: 10 cm) in the two vineyards at four soil depths (10, 20, 60, 100 cm) before and 2/4 weeks of flooding. The samples were divided into two parts and transported to the laboratory on ice on the same day of sampling. One part was stored in −80 °C for microbial analyses, while the other was stored at 4 °C for physicochemical analyses for 1 week. Additionally, water samples for monitoring NO_3_^–^ leaching were sampled during the whole flooding period and reported in our previous study,^28^ but only samples before and 24- and 48-h after flooding were used for the current study. The detailed information of water sampling is provided in the Supporting Information.

### 2.2 Sample and Data Analyses

To achieve our research aim, we performed comprehensive analyses that consisted of (1) molecular analyses of the microbial communities through sequencing of the 16S rRNA gene and quantitative PCR (qPCR) of functional genes, and metagenomic reconstruction of both nitrifier and denitrifier groups, together with the threshold indicator taxa analysis (TITAN) to investigate the ecological niches and environmental controls of the two groups; and (2) field geochemical analyses to monitor NO_3_^–^ leaching and dual-isotope (^15^N and ^18^O) analyses to estimate the *in situ* denitrification activity followed by microcosm-based studies to quantify the net and potential nitrification and denitrification rates at different depths under the Ag-MAR application. The detailed methodology is provided in the Supporting Information. The detailed experiment design and sampling, and data analyses are illustrated as a schematic flowchart in Figure S1.

## 3 Results

### 3.1 Nitrogen Leaching Was More Pronounced after 24 h of Flooding for Recharge

**Figure 1:**
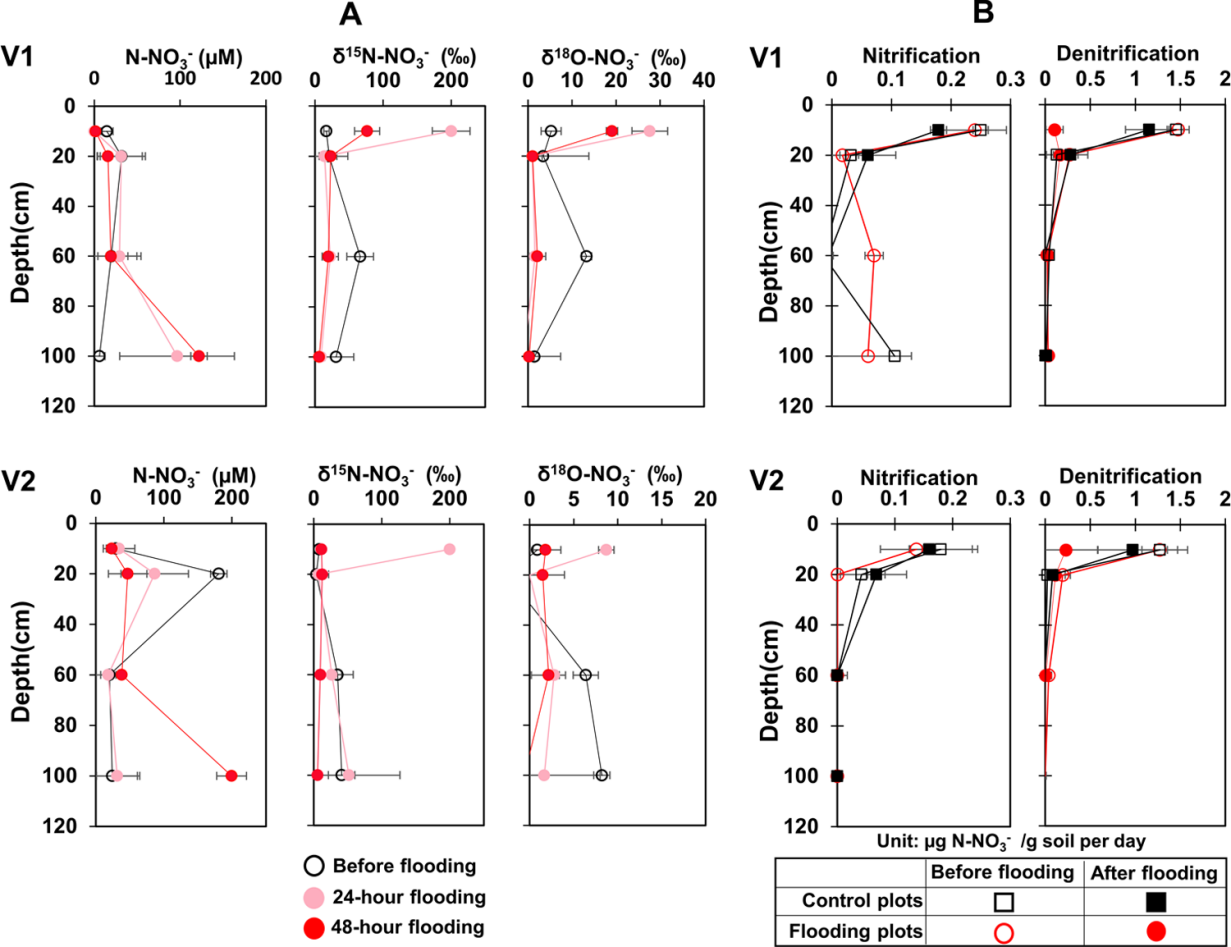
(A) Changes in dual-isotopic fractions (^15^N and ^18^O) of NO_3_^–^ at three time points in the field and (B) net nitrification and denitrification rates measured in the lab soil incubation at four soil depths (10, 20, 60, 100 cm) before and after flooding in two vineyards. V1, large vineyard; V2, small vineyard. Control plots had 6 replicates, and flooding plots had 12 replicates at each depth.

To monitor the effects of flooding on NO_3_^–^ leaching along the soil profile, we measured the NO_3_^–^ concentrations in porewater collected at four depths (10, 20, 60, 100) before and 24- and 48-h after field flooding. The initial mean concentrations of porewater N–NO_3_^–^ at the four measured depths ranged from 6.07 ± 6.53 to 180.67 ± 12.11 μM in the two vineyards, with highest values of 31.88 ± 27.81 and 180.67 ± 12.11 μM at the depth of 20 cm in V1 and V2, respectively. In general, we observed a decrease in porewater N–NO_3_^–^ in the top 60 cm, followed by a striking increase at a depth of 100 cm after 24 or 48 h of flooding. Specifically, within 48 h of flooding, there was a 2–4-fold decrease in the mean concentrations of porewater N–NO_3_^–^ at 20 cm, decreasing from 31.88 ± 27.81 to 16.04 ± 12.73 μM in V1 and from 180.67 ± 12.11 to 46.79 ± 28.23 μM in V2 (Figure 1A). As we anticipated, there was a significant increase in porewater N–NO_3_^–^ (10–20-fold) at the depth of 100 cm. The increase was more pronounced in V2 (from 23.99 ± 4.07 to 199.53 ± 21.95 μM; ANOVA-Tukey’s HSD test with *P* < 0.05) compared to V1 (from 6.07± 0.65 to 122.13± 9.59 μM; ANOVA-Tukey’s HSD test with *P* < 0.05). The average leaching rates of N–NO_3_^–^ after 48 h of flooding at the depth of 100 cm was 58.79 ± 4.85 and 83.94 ± 12.14 μM/day in V1 and V2, respectively. The higher leaching rate of N–NO_3_^–^ in V2 compared to V1 was likely attributed to the higher infiltration rate in V2 (0.171 ± 0.025 m/day) than that in V1 (0.088 ± 0.031 m/day).

### 3.2 Both Net and Potential Rates of Nitrification and Denitrification Were Depth Driven and Decreased after Flooding

We used the combination of field dual-isotopic analyses and laboratory incubation study to determine the effects of flooding on nitrification and denitrification activities before and after 24 and 48 h of flooding. Initially, the porewater δ^15^N–NO_3_^–^ and δ ^18^O–NO_3_^–^ were generally 3–5-fold lower at 0–20 cm than those observed at 60–100 cm. After flooding, changes in these heavy dual isotopes varied across soil depths. For instance, after 24 h of flooding in both vineyards, we found the highest levels of heavy isotopic signatures in the top 10 cm of soil. The average values increased significantly, with δ^15^N–NO_3_^–^ increasing up to 12-fold from 16.64 ± 3.99 to 200 ± 24.73‰ and δ^18^O–NO_3_^–^ increasing up to 5-fold from 5.26 ± 2.29 to 27.66 ± 4.05‰ in V1 (ANOVA-Tukey’s HSD test with *P* < 0.01). Compared to V1, these values increased up to 25 times from 8.0 ± 0.7 to 200 ± 1.36‰ for δ^15^N–NO_3_ and up to 10 times from 0.87 ± 0.46 to 8.71 ± 0.88‰ for δ^18^O–NO_3_^–^ in V2 (ANOVA-Tukey’s HSD test with *P* < 0.01; Figure 1A). However, after 48 h of flooding, the levels of heavy dual-isotopic signatures were lower than 24-h of flooding, reaching only 5-fold enrichment for δ^15^N–NO_3_^–^ and 4-fold enrichment for δ^18^O–NO_3_^–^ in V1, 1.5-fold enrichment for δ^15^N–NO_3_^–^ and 2-fold enrichment for δ^18^O–NO_3_^–^ in V2. At deeper depths of 60–100 cm, we measured, however, 5–8-fold dilutions in both isotopic signatures after 48 h of flooding. In the topsoil, the average enrichment factors for δ^15^N–NO_3_ ranged from εN = −15.34 to −38.97‰ and for δ^18^O–NO_3_^–^, they ranged from εO = −4.17 to −6.14‰ after flooding. While in the deeper soil, these enrichment values showed a narrower range, with εN = −8.04 to −16.68‰ for δ^15^N–NO_3_ and εO = −0.41 to −4.15‰ for δ^18^O–NO_3_^–^. The observed ranges of enrichment factors in our study generally align with the values of microbial denitrification that were summarized in a previous report,^17^ which range from −4 to −30‰ for δ^15^N–NO_3_^–^ and −2 to −18‰ for δ^18^O–NO_3_^–^. Altogether, our results indicated that the denitrification activities were much lower in deeper layers compared to top layers and decreased over time. The microcosm incubation results also demonstrated that microbial activities controlling the fate of N–NO_3_^–^ during flooding, namely net/potential nitrification and denitrification (Figures 1B and S3), were sharply decreased with depth. Net nitrification rates were 8–25 times higher in the topsoil (0–10 cm; 0.13–0.25 μg N–NO_3_^–^/g soil per day) than in the deeper soil (0.01–0.03 μg N–NO_3_^–^/g soil per day), and net denitrification rates were 7–75 times higher in the topsoil (1.3–1.5 μg N–NO_3_^–^/g soil per day) than in the deeper soil (0.02–0.2 μg N–NO_3_^–^/g soil per day). After flooding, denitrification rates were decreased around 10-fold while nitrification was totally inhibited in the topsoil with the measured soil moisture being around 25% (circles in Figure 1B and Figure S2). Conversely, these activities were not significantly altered for controls (squares in Figure 1B). Based on these observations, our hypothesis that nitrification and denitrification activities were stratified with depth was confirmed and their activities decreased over time during the flooding period.

### 3.3 Microbial Community Showed Significant Difference between Depths with High Resistance to Flooding for Recharge

**Figure 2:**
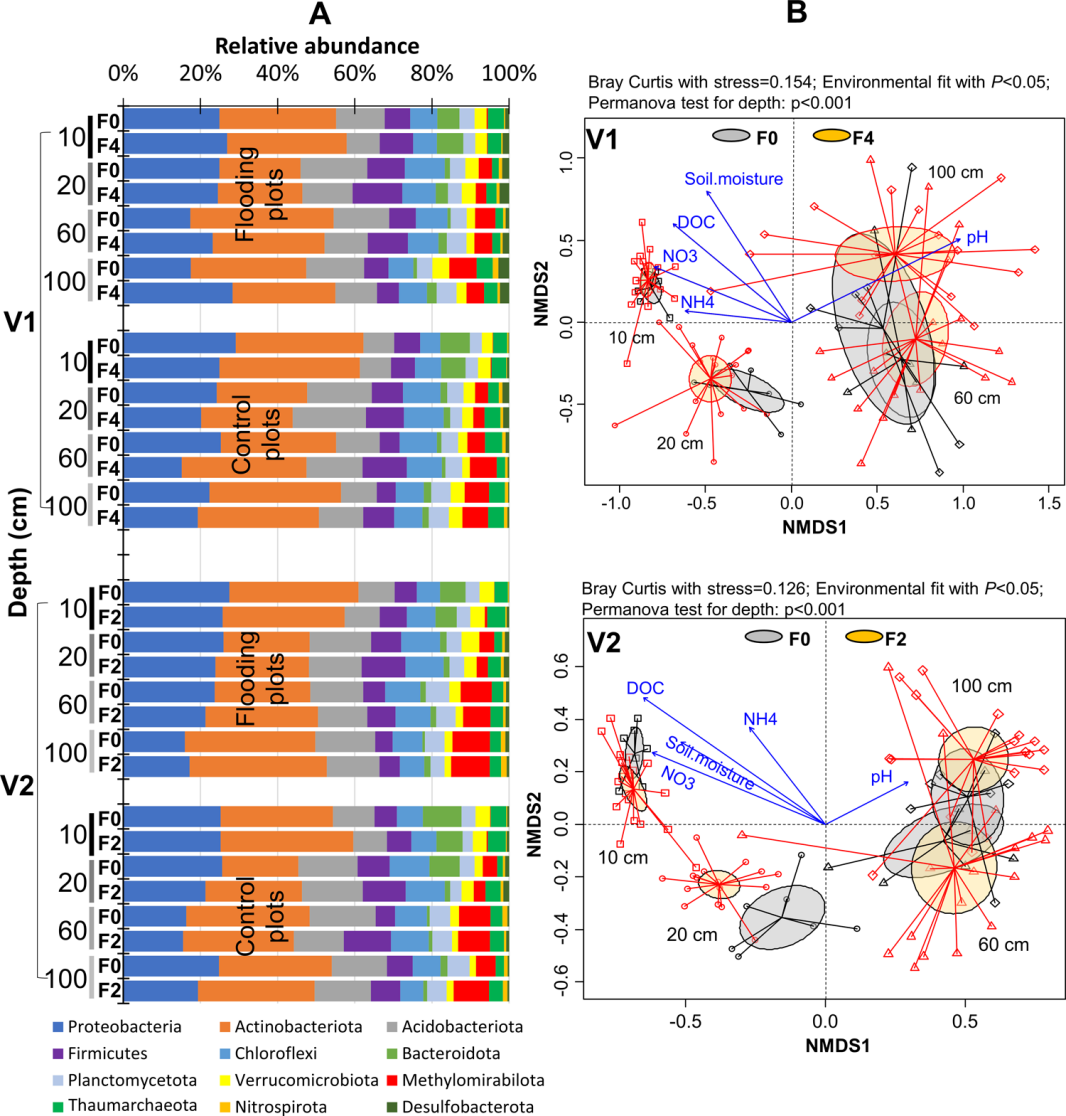
(A) Changes in relative abundance of microbial community (phylum level) and (B) β-diversity (Bray–Curtis distance) at four soil depths (10, 20, 60, 100 cm) before and after flooding in two vineyards. V1, large vineyard; V2, small vineyard. F0, samples before flooding; F2/F4, samples after 2/4 weeks of flooding. Control plots had 6 replicates, and flooding plots had 12 replicates at each depth.

The V4 region of the prokaryotic 16S rRNA gene was sequenced to assess the changes in the composition of microbial communities before and after flooding at the four measured depths. We found that more than 90% of sequences were assigned to the following phyla in both vineyards: *Proteobacteria* (15–28%), *Actinobacteriota* (17–32%), *Acidobacteriota* (8–18%), *Firmicutes* (4–11%), *Chloroflexi* (5–9%), *Bacteroidota* (1–9%), *Planctomycetota* (2–5%), *Verrucomicrobiota* (1–4%), *Methylomirabilota* (0.1–8%), *Thaumarchaeota* (2–4%), *Nitrospirota* (0.2–2%), and *Desulfobacterota* (0.1–2%). With the exception of the increase of *Methylomirabilota* and decrease of *Bacteroidetes* with depth, as shown in Figure 2A, the vertical distribution of most phyla appeared to be arbitrary and without any noticeable patterns. Although the majority of bacteria exhibited minor variations pre and post flooding, *Proteobacteria* and *Bacteroidota* exhibited an enrichment of ∼6.4 to 15.2%, and 1.4 to 1.6%, respectively, at deeper depths (60–100 cm) after flooding, which was possibly attributed to the carbon being carried down to these layers and facilitating their growth (Figure S2). To the contrary, *Acidobacteriota* demonstrated a decrease in relative abundance ranging from 1.4 to 6.3% at all soil depths, and some other phyla, like *Firmicutes*, *Verrucomicrobiota*, *Thaumarchaeota, Gemmatimonadota*, *Desulfobacterota*, *Myxococcota*, exhibited a reduction in relative abundance solely at deeper soils (60–100 cm).

α-Diversity indices (Richness observed ASVs, Shannon and Faith’s phylogenetic diversity) and a β-diversity index (Bray–Curtis distance) were calculated to evaluate the changes in microbial diversities along the soil profile before and after flooding. In comparison to samples collected at topsoil from 10 to 20 cm, flooding led to decreases in both Richness observed ASVs and Shannon diversities at a deeper depth of soil ranging from 60 to 100 cm. However, the observed changes in all α-diversity indices before and after flooding were not statistically significant in either of the vineyards (Figure S4, *T*-test; *P* > 0.05). Unexpectedly, these indices did not show significant decrease with depth either (Figure S4, ANOVA with Tukey’s HSD test; *P* > 0.05). Differences in β diversity that were related to the flooding event were most pronounced at 100 cm soil in V1 (Figure 2B and Table S1, PERMANOVA with *R*^2^ = 0.087, *F* = 1.234, *P* = 0.095) and 20 cm soil in V2 (Figure 2B and Table S1, PERMANOVA with *R*^2^ = 0.114, *F* = 1.55, *P* = 0.08) in comparison with other depths, but we found no statistically significant differences in overall community before and after flooding (Figure 2B and Table S1, PERMANOVA with *R*^2^ = 0.064–0.11, *F* = 0.95–1.55, *P* > 0.05). In contrast to the flooding event, β diversity showed significant differences with depth (Figure 2B and Table S2, PERMANOVA with *R*^2^ = 0.05–0.32, *F* = 4.24–22.08, *P* = 0.001). In addition, intensive agricultural practices homogenized the topsoil and led to a smaller variation in microbial communities at top layers than that at deep layers, with most variation being visible at 100 cm (Figure 2B). The DOC, NH_4_^+^, NO_3_^–^, soil moisture, and pH significantly contributed to the variation and clustering of microbial communities with depth in both vineyards (environmental fit with *P* < 0.05; Figure 2B). Generally, nonsignificant response of microbial community to the soil physicochemical fluctuations (Figure S2) in this study supported that microbial community was overall resistant to the temporal changes in soil conditions triggered by the short-term flooding recharge in the field (i.e., 2–4 weeks of continuous flooding).

### 3.4 Nitrifiers and Denitrifiers Demonstrated Depth-Specific Distribution Patterns

**Figure 3:**
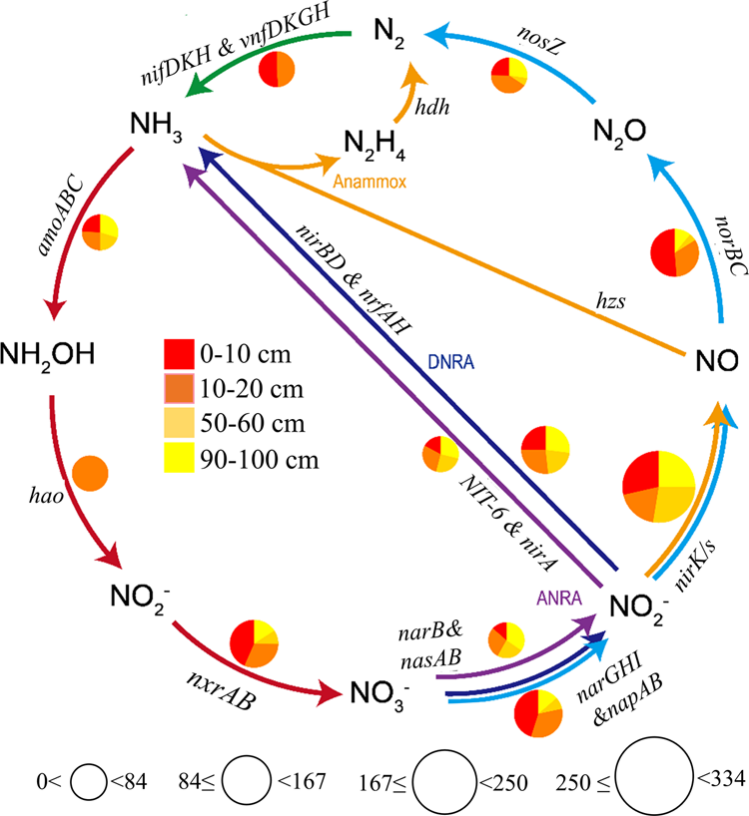
Gene abundance (sequence counts) of nitrogen cycling in different soil depths (0–10, 10–20, 50–60, 90–100 cm) identified by metagenomic sequence. The size of the circle represents the counts and color within the circle represents different depths. Nitrogen fixation pathway: *nifDKH*, *vnfDKGH*; nitrification pathway: a*moABC*, *hao*, *nxrAB*; denitrification pathway: *narGHI*, *napAB, nirK/S, norBC, nosZ*, assimilatory nitrate reduction to ammonium pathway (ANRA): *narB*, *nasAB*, *nirA*, dissimilatory nitrate reduction to ammonium pathway (DNRA): *narB*, *nasAB*, *nirBD*, *nrfAH*; and anaerobic ammonium oxidation pathway (anammox): *hzs*, *hdh*.

The depth-related patterns of all N cycling-related genes were first estimated via metagenomics using the DiTing pipeline (Figure 3), which includes pathways of nitrogen fixation (*nifDKH*, *vnfDKGH*), nitrification (*amoABC*, *hao*, *nxrAB*), denitrification (*narGHI*, *napAB*, *nirK/S*, *norBC*, *nosZ*), assimilatory nitrate reduction to ammonium (ANRA *– narB*, *nasAB*, *nirA*), dissimilatory nitrate reduction to ammonium (DNRA - *nirBD*, *nrfAH*), and anaerobic ammonium oxidation (anammox – *hzs*, *hdh*). Our results showed that the nitrogen fixation pathway was present only in the top 20 cm of soil with equal counts (∼40) at each depth. For the nitrification pathway, we observed that the ammonia oxidation (*amoABC*, *hao*) was the limiting step with lower gene counts (∼80) than that (∼160) of nitrite oxidation (*nxrAB*), while the *hao* gene that controls hydroxylamine oxidation was present only at the depth of 20 cm. Genes for other pathways, namely denitrification, ANRA, and DNRA, were detected for controlling NO_2_^–^/NO_3_^–^ reduction. Among them, the denitrification process (*nirK/S*; ∼330 counts) was dominant over the other two (DNRA with 160 counts and ANRA with 80 counts) as the major controlling factor on NO_2_^–^ reduction in soils, with highest gene counts in the top 10 cm. The *nosZ* gene that controls the last step of denitrification showed fewer gene counts (∼80 counts) than all of the other steps. Based on the above analysis, we found that denitrification rather than DNRA, ANRA, and annamox was identified as the major pathway in controlling NO_3_^–^ removal, and that nitrification was the limiting step in controlling NO_3_^–^ production in our study. Therefore, we further quantified the gene copies for these two processes using quantitative PCR and profiled their taxonomy via metagenomics.

Quantitative PCR was performed to examine the abundance of nitrifiers and denitrifiers along the soil profile using specific primer sets that target the *amoA, nirK, nirS*, and *nosZ* genes (Figure S5). The abundance of the *amoA* gene targeting nitrifiers ranged from 3.32 × 10^4^ to 36.9 × 10^7^ copies/g dry soil on average with decreasing trends with depth at both sites. Across all samples, bacterial *amoA* was 1 order of magnitude less abundant and decreased much more in their abundance (100 times) with depth compared to archaeal *amoA*. The abundance of the *nirK, nirS*, and *nosZ* genes targeting denitrifiers ranged from 3.04 × 10^6^ to 7.54 × 10^9^ copies/g dry soil on average. Similarly, all denitrification genes decreased with depth and *nirK*-denitrifiers were the dominant group that exceeded *nirS*-denitrifiers with 10–100 times at all depths. However, neither the nitrifier nor the denitrifier gene abundance varied significantly before and after flooding (Figure S5; ANOVA with Tukey’s HSD test; *P* > 0.05), which corroborated that microbial groups related to nitrification and denitrification were also resistant to flooding recharge.

**Figure 4:**
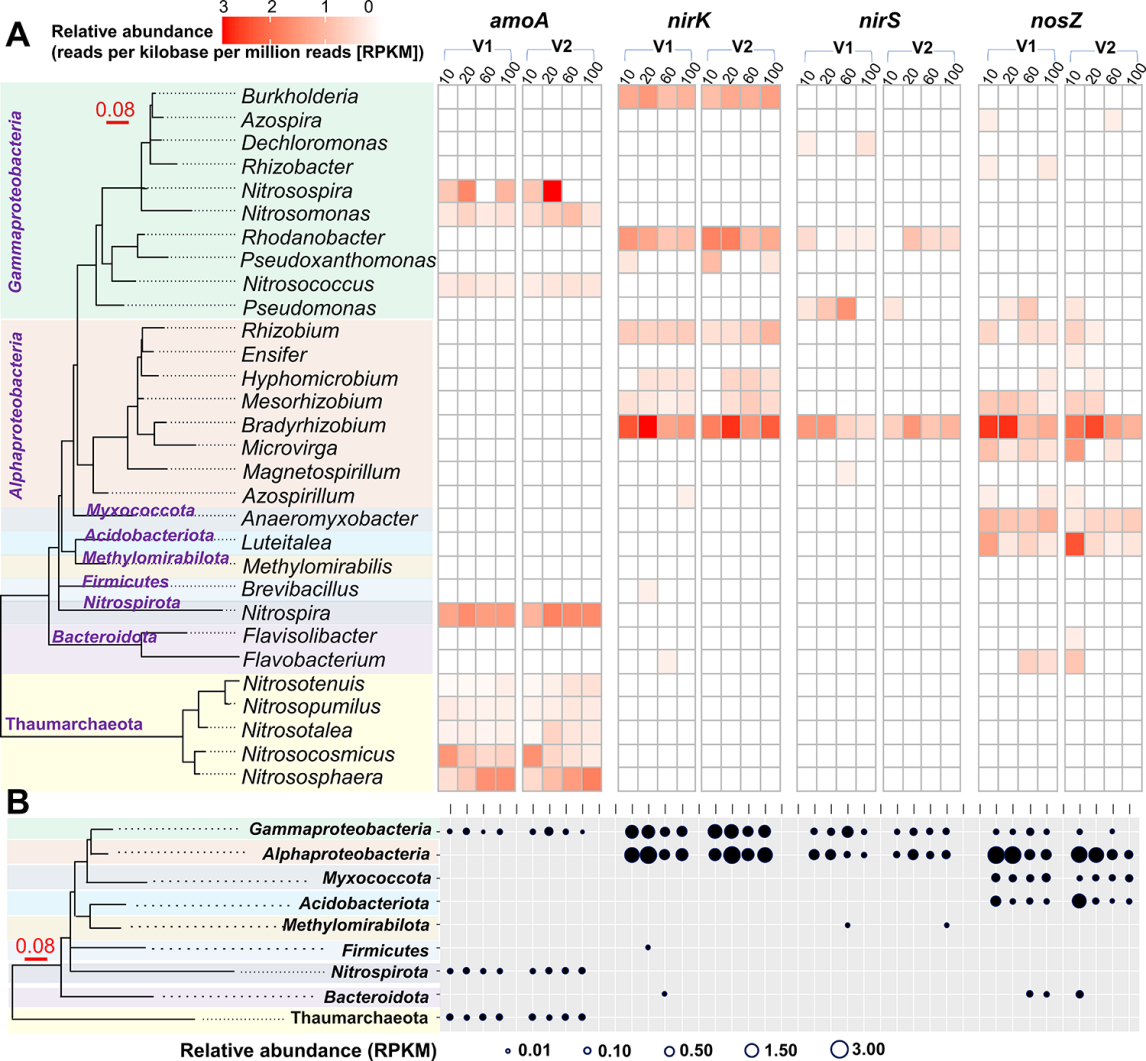
Maximum-likelihood phylogenetic trees (16S rRNA gene based) and heatmaps of the relative abundance of functional genes (*amoA, nirK/S, nosZ*) identified through metagenomic sequence ((A) genus level; (B) phylum level) at four soil depths (10, 20, 60, 100 cm) in two vineyards. V1, large vineyard; V2, small vineyard.

Taxonomic profiling of the *amoA, nirK, nirS*, and *nosZ* sequences was further obtained from metagenomic analysis in this study (Figure 4A,B), and we identified 30 microbial genera that contained either *amoA* or *nirK/S*, *nosZ* genes. These nitrifiers and denitrifiers belonged to *Gammaproteobacteria* (10 genera), *Alphaproteobacteria* (8 genera), *Thaumarchaeota* (5 genera), *Bacteroidota* (2 genera), *Nitrospirota* (1 genus), *Myxococcota* (1 genus), *Acidobacteriota* (1 genus), *Firmicutes* (1 genus), and *Methylomirabilota* (1 genus). Notably, the *amoA* gene was detected in phyla of *Thaumarchaeota*, *Nitrospirota*, and *Gammaproteobacteria* (0.03–0.12 PPKM; Figure 4B) and was mostly allocated to genera of *Nitrosocosmicus, Nitrososphaera*, *Nitrospira* and *Nitrosospira*. In contrast to *Nitrososphaera* and *Nitrospira*, the relative abundance of the *amoA* gene in *Nitrosocosmicus* was higher in the topsoil and decreased with depth (Figure 4A). The genes (*nirK/S*) related to nitrite reduction were mainly present in *Gammaproteobacteria* and *Alphaproteobacteria* with higher *nirK* (0.76–3.05 PPKM) than *nirS* (0.05–0.82 PPKM; Figure 4B). The *nosZ* gene (0.02–3.00 PPKM) encoding for a nitrous oxide reductase, however, was found not only in *Gammaproteobacteria* (0.04–0.22 PPKM) and *Alphaproteobacteria* (dominant with 0.04–3.00 PPKM) but also in *Myxococcota* (0.05–0.42 PPKM), *Acidobacteriota* (0.02–1.93 PPKM), and *Bacteroidota* (0.08–0.28 PPKM), groups that are not known to harbor the nirK/S genes. Among all of the denitrifiers, the *Bradyrhizobium* group was the most abundant denitrifiers that harbored *nirk/S* and *nosZ* genes with lower abundance at deeper depths (Figure 4A). Altogether, the qPCR and metagenomic analyses conveyed that both nitrifiers and denitrifiers exhibited depth-related distribution patterns and possessed different gene profiles.

### 3.5 Nitrifiers and Denitrifiers Occupied Different Environmental Niches

**Figure 5:**
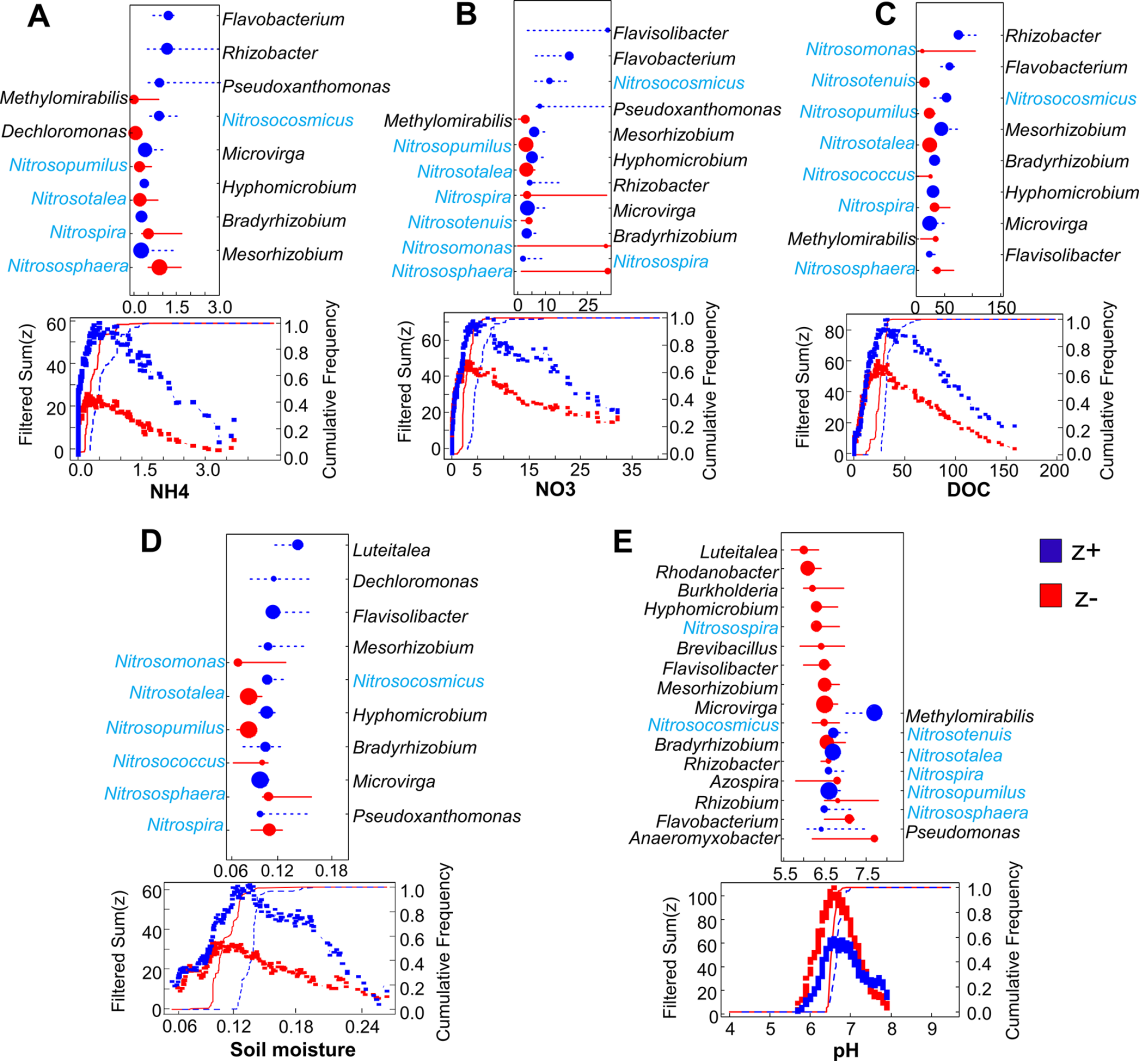
Threshold indicator taxa analysis (TITAN) of nitrifiers (blue color) and denitrifiers (black color) in response to (A) NH_4_^+^ (μg/g dry soil), (B) NO_3_^–^ (μg/g dry soil), (C) DOC (μg/g dry soil), (D) soil moisture, and (E) soil pH. Only significant (purity >0.95, reliability >0.95, *P*-value < 0.05) indicator taxa are plotted in these figures. Dark blue symbols represent positive (*z*^+^) indicator taxa, whereas red represents negative (*z*^–^) indicator taxa. The size of the symbols is in proportion to the *z* scores. Horizontal lines overlapping each symbol represent the 5th and 95th percentiles among 500 bootstraps with a total of 36 replicates at each depth. The lower part of each panel shows filtered values of sum(*z*^–^) and sum(*z*^+^) along the environmental gradient.

The threshold indicator taxa analysis (TITAN) was performed to evaluate how nitrifiers and denitrifiers responded to the changes in the environmental gradients with depth. Significant (purity >0.95, reliability >0.95, *P* < 0.05) indicator taxa are plotted in Figure 5. The negative responders (*z*^–^) are shown on the left side with red color, while positive responders (*z*^+^) are on the right side in blue color. Archaeal nitrifiers harbored broader environmental niches when compared to bacterial nitrifiers. In general, most nitrifiers (light blue names) had an opposite response to the changes in the environmental gradients compared to denitrifiers (black names), indicating that these two groups occupied different environmental niches with depth. To be specific, most nitrifiers decreased, while denitrifiers increased as NH_4_^+^, NO_3_^–^, DOC, and soil moisture increased, and pH decreased with depth (Figure 5). However, there were some exceptions; *Nitrosocosmicus* (nitrifiers) always fell into the similar response trend as denitrifiers did, and on the other hand, *Methylomirabilis*, a known denitrifier, had similar response to the gradients with most nitrifiers. Meanwhile, environmental thresholds (change points) showed that abrupt changes (sharp increase or decrease) occurred in both nitrifiers and denitrifiers. In both groups, most taxa showed general change points (95% confidence interval, CI) at an NH_4_^+^ concentration of ∼0.55 μg/g dry soil, a NO_3_^–^ concentration of ∼6.31 μg/g dry soil, a DOC concentration of ∼32.35 μg/g dry soil, the soil moisture of ∼13%, and the pH of ∼6.7. Among all of the nitrifiers, *Nitrosopumilus* was identified as the most sensitive lineage that negatively responded to the increasing gradients of NH_4_^+^ and NO_3_^–^ with lowest changing points of 0.45 μg/g dry soil of NH_4_^+^ and 3.43 μg/g dry soil of NO_3_^–^, respectively; while *Nitrososphaera* was identified as the least sensitive genus with highest changing points of NH_4_^+^ with 1.54 μg/g dry soil and NO_3_^–^ with 32.71 μg/g dry soil, respectively (Figure 5A,B). In contrast, *Nitrosocosmicus* was highly tolerant to NH_4_^+^ and NO_3_ as it positively responded to the increased gradients of NH_4_^+^ and NO_3_^–^. The dominant *nirK/S*-type denitrifier *Bradyrhizobium* primarily and positively responded to the increased gradient of NO_3_^–^ with lowest changing point at 6.94 μg/g dry soil (Figure 5B) among all of the denitrifiers and to a lesser extent responded to the increased gradients of DOC and soil moisture (positively; Figure 5C,D), as well as pH (negatively; Figure 5E). The dominant *nosZ*-type non-denitrifiers *Luteitalea*, however, were primarily controlled by both soil moisture (positively; Figure 5D) and pH (negatively; Figure 5E).

## 4 Discussion

In this study, we combined field and laboratory experiments to provide multifaceted evidence of microbial controls on the fate of NO_3_^–^ during leaching under Ag-MAR events. The majority of microbial groups showed minor variations, resulting in no observed significant changes in the whole microbial composition before and after flooding as illustrated in Figure 2B, which is in agreement with a previous study.^17^ Our study, however, expanded on this by examining alterations at different depths and showed that the microorganisms in deeper soil depth were more susceptible to flooding than those at the surface 0–10 cm. Particularly, we observed the largest enrichment of *Proteobacteria* at depths of 60 and 100 cm (Figure 2A), which has been documented in a recent Ag-MAR study at the depth with carbon-rich permeable reactive barrier (PRB),^18,24^ suggesting that this phylum relies on carbon availability for the growth. Additionally, the depth-specific flooding impacts disclosed that the relative abundance of some minor groups was decreased (Figure 2A), leading to a non-statistically significant decrease in α diversities (e.g., Richness observed ASVs and Shannon diversities in Figure S4) in deep soils (60–100 cm). Concomitant decreases in the total number of unique OTUs (Richness) after flooding have been reported in a previous study.^18^ The different compositional changes after flooding seem to have no significant impact on the functional pools related to nitrification and denitrification across depths, as shown in Figure S5 (e.g., *amo*A, *nirK/S*, *nosZ*). This disconnection between the changes in the whole community and functional pools at different depths has not been reported in Ag-MAR studies before.

Our results further showed that the changes in microbial community and functional genes were driven more by depth rather than the flooding event (Figures 2 and 3 and Tables S1 and S2). Previous studies also demonstrated that the overall microbial communities, and particularly to this study, archaeal nitrifiers, and *nirS*-type denitrifiers were very resistant to short-term dry–wet processes.^29−31^ It has been suggested that the changes in microbial communities and functional groups during soil wetting processes are not only related to shifts in physicochemical properties but also influenced by wetting duration and seasons,^29,32^ ecosystem types, and soil textures,^33,34^ as well as to a large extent by nutrient acquisition strategies and physiologies of microbes.^31,35,36^ Additionally, Emsens et al.^32^ conducted a long-term rewetting study (years to decades) in peatland soil, where it was found that the changes in microbial community driven by depth were more pronounced than those observed according to the drainage status. The depth-driven patterns of microbial communities and functions have been reported for different ecosystems including peatlands,^32^ forests,^37,38^ grasslands,^37,39^ and agricultural ecosystems,^40,41^ as well as aquatic ecosystems^42^ and floodplain sediments.^23^ Our results, however, expand on previous findings as it reveals the combined influence of soil carbon content (DOC), nutrient status (NH_4_^+^, NO_3_^–^), and soil physical properties (e.g., soil moisture and pH) that significantly contributed to the depth-driven patterns of microbial community (environmental fit with *P* < 0.05; Figure 2B). Previous studies argued that the dominant effects of depth on the whole community structure were most likely induced by energetic constraints related to aforementioned soil physicochemical conditions with depth.^23,32,37^ There is evidence that this is happening in our system as DOC concentrations were significantly higher in 0–10 cm depth in comparison with other soil depths (Figure S2).

Given that the soil depth also affected the patterns of functional groups, we further employed metagenomic analysis and TITAN to investigate the depth-specific profiles and environmental niches of nitrifiers and denitrifiers that influenced NO_3_^–^ as it leached through the soil profile. In line with our first hypothesis, we found that nitrifiers harbored different environmental niches (Figure 5) and showed varying abundance with depth as confirmed by the relative abundance of the *amoA* gene through metagenomics (Figure 4). For example, *Nitrosocosmicus* was the only nitrifier that dominated in the topsoil, which was in contrast with other nitrifiers that dominated in deeper soils like *Nitrososphaera, Nitrosospira*, and *Nitrospira*. Accordingly, TITAN established that *Nitrosocosmicus* occupied very different environmental niches and had much higher tolerance to NH_4_^+^ concentrations (>1.67 N-NH_4_^+^ mg/kg) in comparison with other nitrifiers (<1.2 N-NH_4_^+^ mg/kg; Figure 5A). This agrees with the findings that the microorganism *Nitrosocosmicus franklandianus* has the lowest affinity to ammonia among all cultivated archaeal nitrifiers, which was similar to some bacterial nitrifiers and greatly contributed to its high tolerance to NH_4_^+^ concentration in the soil.^43−45^ However, an ammonium-limited enrichment of Ca. *Nitrosocosmicus* was recently recovered in Florida fertile soils,^46^ inferring that other environmental factors or metabolic-related physiology may also significantly affect their survival in the soil. TITAN further showed that different nitrifiers had distinct changing points within each environmental gradient (Figure 5), while the response of the *Nitrosocosmicus* lineage to these environmental gradients, including NO_3_^–^, DOC, pH, and soil moisture, was opposite to that of most nitrifiers. Nitrifiers with different niches of NO_3_^–^, DOC, pH, and soil moisture have been reported in numerous previous studies,^23,31,35,45,47,48^ while no study fully reported on the environmental range, identified here as changing points, for nitrifiers and the positive response of *Nitrosocosmicus* to most of these factors in agricultural soils. Attributing to the different niches from other groups, the *Nitrosocosmicus* group was also reported to possess 3–5-fold higher nitrification rate than the *Nitrososphaera* group in both soils^49^ and laboratory cultures.^45,50^ Collectively, the dominant effect of soil depth on nitrifiers was not only imposed by energetic restriction related to NH_4_^+^ and O_2_ but also reflected by other physiological traits that have been observed in marine systems,^51^ yet to be fully explored in soils^50^ and sediments.^23,52^

Recognized as important denitrifier groups, *Burkholderiales, Bradyrhizobiaceae*, as well as *Pseudomonas* and *Paracoccus* have all been widely reported to control the fate of NO_3_^–^ in agricultural soils;^17,53^ however, gene profiles of each individual group at different soil depths are yet to be systematically evaluated. Our study investigated the depth profiles of the genes *nirK, nirS*, and *nosZ* presented in each denitrifier group via metagenomics. We observed that *Bradyrhizobium* (*Alphaproteobacteria*) was the most abundant genus and the only group that harbored all three genes (*nirK, nirS*, and *nosZ*) and showed a general decreasing trend with depth. Instead, other denitrifiers either lacked (*Burkholderia* and *Rhodanobacter*) or only contained (*Microvirga, Anaeromyxobacter*, and *Luteitalea*) *nosZ* genes (Figure 4A). In fact, *Bradyrhizobium, Pseudomonas*, and *Paracoccus* were the three most prevalent but few genera that harbored all three genes,^53−55^ while the *Anaeromyxobacter* group was previously identified as the major *nosZ* non-denitrifier without *nirK* and *nirS* genes in different soils.^56,57^ The incomplete gene profiles were described in a vast number of denitrifiers by a profusion of studies,^56−59^ yet this is important because the process of denitrification is expected to be more thermodynamically efficient when the microorganism only regulates one step rather than mediate multiple steps at a time.^60^ A recent study further revealed that some of the *Bradyrhizobium* strains preferred N_2_O (*nosZ* controlled) over NO_3_^–^ reduction (*nirK/S* controlled), resulting in an ∼6-fold lower rate in NO_3_^–^ reduction and 25-fold lower rate in NO_2_^–^ reduction when compared with *Paracoccus* strains.^61^ Therefore, this could be one of the explanations on low NO_3_^–^/NO_2_^–^ removal efficiency in our agricultural soil dominated by the *Bradyrhizobium* group.

The differential partitioning among the denitrifiers did not only occur in the gene profiles of each group but was also reflected in the environmental preference between *nirK* and *nirS* types. Consistent with other studies,^25,62^ our results also showed that *nirK*-type denitrifiers were, in general, more abundant than *nirS*-type denitrifiers with lower relative abundances of both groups when approaching the deeper depths (Figures 3, 4, and S5). Another important implication of our study is that most denitrifiers showed a distinct range of environmental responses, as evidenced by TITAN, with a positive response to the increases in soil NO_3_^–^, DOC, and soil moisture (Figure 5). Previous isolated aerobic denitrifiers, including *Bacillus*, *Dechloromonas*, *Flavobacterium*, *Mesorhizobium*, and *Pseudomonas*,^63−67^ were also found in our soils, but with a dominance of the *nirS*-type *Pseudomonas* group. Accordingly, the *nirS*-type *Pseudomonas* group had a higher O_2_ tolerance than *nirK*-type (e.g., *Enterobacter* strain I-25 and *Achromobacter* strain I-49) and was also corroborated by AbuBakr and Duncan.^62,63^ Together, these observations thus support the idea that the effects of depth-related carbon and NO_3_^–^ availability overrode the effects of O_2_ levels on soil denitrifiers as reported in many studies.^25,53,68^ Soil pH was also shown as a key factor in controlling the niches of the denitrifiers.^58,69^ Compared with the *nirS*-type groups like *Pseudomonas* that positively responded to the increases in pH with a changing point of 7.8, the *nirK*-type groups including *Burkholderia, Rhodanobacter, Rhizobium, Hyphomicrobium*, and *Mesorhizobium* negatively responded to the increases in pH with a changing point lower than 7.0 (Figure 5E). Bowen et al.^70^ supported our results in reporting that the *nirS*-type denitrifiers were more active in high pH soils, while the *nirK-*type groups showed higher activity in soils with low pH. In most cases, low pH (<3.0), however, would greatly decrease denitrification activity by inhibiting transcriptionally active denitrifiers, with a particular delay of N_2_O reduction by postponing *nosZ* expression.^58,69,70^

Regarding the microbial activities associated with NO_3_^–^ leaching at different depths during the flooding period, we found that both net and potential microbial activities related to NO_3_^–^ production and consumption (Figures 1B and S3) followed a sharp decreasing trend with depth, which was consistent with a few previous studies.^16,25^ We also observed increased denitrification activities after 24 h, but the increment dropped after 48 h of flooding in the topsoil (Figure 1A), which partially agreed with a previous report that the denitrification rate increased after soil wetting in agricultural ecosystems.^70^ Hu et al.^71^ further pointed out that the cumulative potential for denitrification increased linearly within the first 6.5 to 24 h and plateaued before 72 h of flooding peatland soils due to the gradual depletion of substrates and microbial competition with time. Interestingly, the net *in situ* microbial activities (Figure 1B) before and after flooding were rarely detected at the deeper layers below 60 cm even with high abundance of functional genes in both vineyards (Figure 3), inferring that the microbial activities in deeper soils were much more limited by substrates rather than the functional gene pool. Specifically, nitrification rates were limited by ammonium, while denitrification rates were limited by carbon availability in deeper soils based on the profiles of NH_4_^+^ and DOC concentrations (Figure S2), which was in line with numerous previous studies.^17,18,25,72^ Meanwhile, we also observed that a very high concentration of NO_3_^–^ (around 200 μM), which was 10–20 times the initial soil residual NO_3_^–^ content, leached down to 1 m depth after 48 h of flooding in both vineyards, where denitrification activities decreased from 1.5 to 0.1 μg/g dry soil per day in above 0.2 m but were barely detected in soils below 0.6 m. Altogether, these results again indicated that NO_3_^–^ removal was rather constrained during flooding through denitrification activities that were mostly dominated by the *Bradyrhizobium* group in soil even under a low infiltration rate (<0.18 m/day), which agreed with the study of Gorski et al.^17^ with a similar infiltration rate (∼0.17 m/day). As for the effects of infiltration rates, several previous studies summarized that NO_3_^–^ removal only occurred when vertical infiltration rates were <0.7 ± 0.2 m/day in native soils with high removal efficiency falling into a range of 0.2–0.4 m/day.^18,24^ Schmidt et al.^26^ inferred that the redox conditions at very high infiltration rates were not conducive to denitrification, and this may be particularly true for our soil system, which was dominated by the strictly anaerobic denitrifiers (*Bradyrhizobium*). Nevertheless, we still cannot exclude other factors that impact nitrate removal, for example changes in trace metal availability during flooding,^6^ which has been shown to significantly affect denitrification in both laboratory cultures^73−75^ and environmental samples.^76−78^ Our study represents initial and novel efforts to understand the factors controlling NO_3_^–^ removal during Ag-MAR events. We recommend future research to investigate these complex interplays related to microbial controls on NO_3_^–^ leaching with Ag-MAR application.

Our results provided compelling evidence that the microbial community exhibited a high resistance to short-term Ag-MAR events, while microbial activities associated with the processes of nitrification and denitrification were spatially distinct and decreased over time during the flooding period. Our study further suggests wetting the soil to near or above field water-holding capacity moisture to decrease nitrification while promoting denitrification to draw down the nitrate pool prior to flooding.

## Data Availability

The paired-end Illumina 16S rRNA sequence, raw metagenomic sequence for all of the samples in this study were submitted to Sequence Read Archive (SRA) under BioProject PRJNA844995.

## Supporting Information Available

Additional experimental details, materials, and methods, including sampling, geochemical and molecular analyses, as well as data processing. Schematic flowchart of the experiment design, sampling and data analyses (Figure S1); soil physicochemical profile (Figure S2); potential nitrification and denitrification activities (Figure S3); changes in the α diversities (Figure S4); and functional gene abundance (Figure S5). Summary of the Permanova test of the β diversity (Tables S1 and S2) (PDF)
