# Supplementary material for: Molecular and Dual-Isotopic Profiling of the Microbial Controls on Nitrogen Leaching in Agricultural Soils under Managed Aquifer Recharge
Source: Environ Sci Technol. 2023 Jul 19;57(30):11084–95. doi: 10.1021/acs.est.3c01356 (PMC10399200; doi:10.1021/acs.est.3c01356)
Supplement: Supplementary file 1 — es3c01356_si_001.pdf [file es3c01356_si_001.pdf]

Supporting Information

Molecular and Dual-Isotopic Profiling of the Microbial Controls on Nitrogen Leaching in Agricultural Soils under Managed Aquifer Recharge

Laibin Huang[1], Elad Levintal[1], Christian Bernard Erikson[1], Adolfo Coyotl[1], William R. Horwath[1], Helen E. Dahlke[1], Jorge L. Mazza Rodrigues[1, 2*]

[1]Department of Land, Air, and Water Resources, University of California, Davis, CA, 95616, USA

[2]Environmental Genomics and Systems Biology Division, Lawrence Berkeley National Laboratory, Berkeley, CA, 94720, USA

Running Title: Taxa and depth-specific partitioning of soil microbiomes

Synopsis: Conducting groundwater recharge in agricultural systems requires understanding of the biological controls of nitrate leaching and nitrogen cycling processes.

[*]**Corresponding author:** Jorge L. Mazza Rodrigues (jmrodrigues@ucdavis.edu)

Number of Pages: 17

Number of Figures: 5

Number of Tables: 2

2. MATERIALS AND METHODS

2.1. Physicochemical analyses. The soils stored at 4°C were used for determining the soil water content (WC) by weight loss after drying 20 g of wet soil at 105°C for 24 hours. Soil pH was measured in a suspension of 1:5 soil/water (m/v) with a pH meter (Hach Company, Loveland, CO, USA). NO_2^-/NO_3^- and NH_4^+(mg kg $^{-1}$) were extracted using 0.5 M K_2SO_4 and then analyzed by colorimetry with an UV-1280UV-VIS Spectrophotometer (Shimadzu Scientific Instruments, Pleasanton, CA).[1, 2] Dissolved organic carbon (DOC, mg kg^{-1}) was measured with the multi-N/C 3100 TOC/TN analyzer (Analytik, Jena, Germany). Analysis of Variance (ANOVA) with Tukey's HSD test was used to identify the significant differences of the changes in chemicals before and after flooding.

2.2. DNA extraction and amplicon sequencing. Total soil DNA was extracted from ~0.30 (0-20 cm soil) or 0.50 g (60-100cm) of wet soil using the DNeasy Powersoil kits following the manufacturer's instructions (Qiagen, CA, USA). The concentration of DNA was assessed by Qubit 3.0 (Thermo Fisher Scientific, CA, USA) with higher values at 0-20 cm (30-50 ng//µl) and lower values at 60-100 cm (1-10 ng/µl). DNA was viewed using 1.5% agarose gel electrophoresis for quality assessment and stored at -80°C for downstream analyses. The primer sets of 515F[3] and barcoded 806R[4] modified from Caporaso et al.[5] were used to amplify the V4 region (~380bp) of the prokaryotic 16S rRNA gene. Triple Polymerase Chain Reactions (PCRs) with 20 µl mixture were conducted for each sample, which contained 10 µl of 2x Phusion Hot Start II High-Fidelity PCR Master Mix (Thermo Fisher Scientific, USA), 1 µl of each primer (1 µM), 2 µl of template DNA (~5 ng/µl) and 6 µl of PCR grade water. The reaction conditions were set up with an initial denaturation at 98°C for 30 s, 30 cycles of denaturation at 98°C for 15 s, annealing at 50°C for 30 s, and elongation at 72°C for 15 s, followed by a final extension at 72°C for 10 min and then

holding at 4°C. Triplicates of PCR products of same sample were combined and quantified by Qubit 3.0 (Thermo Fisher Scientific, CA, USA). Each sample was pooled with equimolar concentrations (100 ng/each sample) in a single sterile tube and purified using a MoBio UltraClean PCR Clean-Up Kits (MoBio Laboratories Inc., CA, USA) following the manufacturer's instructions. The combined sample was sequenced on the Illumina MiSeq platform (paired end with 2×250 bp) at the DNA Technologies Sequencing Core of the Genome Center at the University of California, Davis (UC Davis).

2.3. Amplicon sequence analyses. A total of 8,017,740 paired-end raw sequences were obtained for 200 samples from the Illumina MiSeq platform with a range from 11,505 to 81,391 reads per sample (average of 40,493 and median of 41,006 reads per sample). Raw reads were imported to the QIIME2 [6] and trimmed to 196 bases for the forward reads and 172 for the reverse reads with a Qscore $\geqslant$ 20, and then chimeric screened via 'DADA2' package.[7] A total of 3,055,783 reads passed quality control and chimeric screening, with an average of 15,433 reads per sample. The feature table with amplicon sequence variants (ASVs) were also obtained via the DADA2 denoise-paired step. All ASVs were aligned using the MAFFT program,[8] and a tree was generated via the phylogeny fasttree step.[9] The ASV table, tree file and mapping file, served as the main inputs for the taxonomy assignment against the SILVA 138 database.[10]

2.4. α- and β-diversities measurements. To assess how the microbial diversity changed before and after flooding, α- (observed ASVs, Shannon, and Faith's phylogenetic diversities) and β-diversity indices (Bray-Curtis distance based non-metric multidimensional scaling, NMDS) were obtained using both the 'phyloseq'[11] and 'vegan' packages in R v.4.0.2[12] at an even sequence depth (10,000 reads) for all samples. The environmental factors that significantly fitted the NMDS model were identified by a permutational test using the 'vegan' package in R v.4.0.2.[12] The permutational

multivariate analysis of variance (PERMANOVA) was tested for the significance of β-diversity between different depths and treatments via the 'pairwiseAdonis' package in R v.4.0.2.[13] Analysis of Variance (ANOVA) with Tukey's HSD test was used to identify the significant differences of the α-diversities and relative abundances of microbial compositions before and after flooding.

2.5. Metagenome sequencing and analyses. We did not find any significant differences in functional genes (as depicted in Fig.S5) and community composition (as illustrated in Fig.2) pre and post flooding. Conversely, we observed noticeable variations solely across different soil depths (as illustrated in Fig.2). Therefore, we opted to select two soil profiles (10, 20, 60, 100 cm) with a total of 8 samples collected before flooding for metagenomic analysis, using the NovaSeq Illumina platform with 151 bp paired-end reads in the DNA Technologies Sequencing Core of the Genome Center at the UC Davis. A total of 421,914,046 paired-end raw reads were retrieved with average of 52,739,255 for each sample. Raw reads were filtered and trimmed using the fastp program[14] with Q score of 35 and minimum length of 140 bp. A total of 348,472,018 reads passed quality control with an average of 43,559,002 reads per sample. Metagenomic taxonomy profiling was conducted using kraken 2.0 [15] then summarized via bit program.[16] Filtered raw reads were first used to evaluate gene abundances of whole N cycling processes using the DiTing software pipeline.[17] Based this analysis, we found that denitrification was dominant over DNRA and annamox as the major pathway in controlling NO_3^- removal, and that nitrification was the limiting step in controlling NO_3^- production. Therefore, we focused on analyzing microbial communities related to denitrification and nitrification processes. To achieve this aim, high-quality short reads ($Q > 35$ with length longer than 140 bp) were aligned against the manually curated functional databases using "Bowtie 2" with the global alignment method.[18] The reference databases obtained from function gene repository (http://fungene.cme.msu.edu/) included archaeal and bacterial

ammonia monooxygenase alpha subunit (*amoA;* 41,712 sequences for bacterial *amoA;* 36, 837 sequences for archaeal *amoA*), nitrite reductase (*nirK and nirS;* 4,535 sequences for *nirK*; 20,735 sequences for *nirS*), nitrous oxide reductase (*nosZ;* 4,047sequences for nosZI; 4234 sequences for *nosZII*). These sequences cover ammonia-oxidizing archaea and bacteria belonging to *Thaumarchaeota* (e.g., *Nitrosocosmicus*, *Nitrososphaera*, *Nitrosotenius*, *Nitrosopumilus*, *Nitrosotalea*) and *Gammaproteobacteria* (e.g.,*Nitrosomonas*, N*itrosospira*, *Niotroscoccus*) and *Nitrospirota* (e.g., *Nitrospira*); the database also covers denitrifiers in phyla of *Alphaproteobacteria*, *Bacteroidota*, *Myxococcota*, *Acidobacteriota*, *Firmicutes*, and *Methylomirabilota*. The full taxonomy name for each sequence hit was extracted using "taxize" package in R v.4.0.2.[19] The relative abundance of the target gene was determined using normalized target reads (counts divided by total reads in each sample and functional gene length) with the unit of reads per kilobase per million reads (PPKM).[20]

2.6. Phylogenetic trees and threshold indicator taxa analysis. Phylogenetic trees were constructed for the genera that harbored nitrification and denitrification genes using 'FastTree' program [19] with default parameters and the trees were visualized using the 'phyloseq' and 'ggtree' packages in R v.4.0.2.[11, 21] Furthermore, the threshold indicator taxa analysis (TITAN) was used to explore the changing point of the relative abundance of these genera along the environmental gradients via 'TITAN2' package in R v.4.0.2.[22] TITAN is a sensitive and precise method for assessing community changing thresholds by detecting abrupt changes in taxa distributions along an environmental gradient over space or time. [22]

2.7. Quantitative polymerase chain reaction (qPCR) of functional genes. Copy numbers of functional genes for nitrification (*amoA*) and denitrification (*nirK/S, and nosZ*) were estimated using a SYBR green-based qPCR in the BioRad CFX Connect real-time PCR system (Bio-Rad

Laboratories, Hercules, CA, USA). The PCR mixture contained 10 µl of SsoAdvanced Universal SYBR Green Supermix (Bio-Rad Laboratories, Hercules, CA, USA), 1 µl each of forward and reverse primers from stock solutions (0.5 µM of final concentration for each primer) and 2 µl of DNA template (0.25 ng/µl of final concentration) in a final volume of 20 µl. Previously described primers and PCR conditions were employed for quantification of genes of *amoA*[23, 24] and *nirK/S*, *nosZ*.[25] All qPCR runs were followed by an image capture step (15 s at 83°C) after a final extension step of each cycle. When the PCR amplification was completed, a melt curve analysis was conducted by increasing the temperature from 60 to 95°C in 0.5°C increments every 10 s to verify that nonspecific amplification did not occur. qPCR standards were made by cloning the target gene fragment using the TA Cloning™ Kit, with pCR™2.1 Vector and One Shot™ TOP10 chemically competent *Escherichia coli* (Thermo Fisher Scientific, Waltham, MA, USA). Seven orders of 10-fold serial dilutions of linearized plasmid DNA were run for each qPCR to generate standard curves. PCR amplification efficiencies were between 83.7% and 99.9% with $R^2 \geqslant 0.995$. The gene copy number was calculated based on the previous study.[26]

2.8. Nitrification and denitrification incubation. Net and potential nitrification were determined using the aerobic incubation procedure in the lab following modified methods described by Verchot et al.[27] Briefly, a 15 g field moist soil sample was placed in a 50-ml falcon tube and closed with a perforated cap to allow gas exchange while minimizing evaporation for the 7-day incubation at room temperature (25°C) under aerobic conditions. One ml of 1.5 mM NH_4Cl was added to 15 g soil for the potential nitrification incubation. Net and potential denitrification rates were performed for each sample using a modified method of Petersen et al.[28], with 15g of moist soil in 100 ml serum bottle incubated for 7 days at 25°C under anaerobic conditions. Five ml of combined 5 mM glucose and 5 mM KNO_3 was added to 15g of soil for the potential denitrification incubation

based on background NO_3^- concentrations. To obtain anaerobic conditions, the headspace was flushed with N_2 gas for 10 minutes and equilibrated with atmospheric pressure using a glass syringe afterwards. The changes in NO_3^- concentrations at initial and 7-day incubated samples were analysed for determining both nitrification (static incubations not slurry rates) and denitrification rates. To extract soil NO_3^-, 0.5 g soil was subsampled before and after incubation with 1 ml of 0.5 M K_2SO_4 solution and shaken for 1 h on an orbital shaker and centrifuged to allow soil particles to settle. The supernatant was filtered through a 2.5 μm Whatman filter paper (MilliporeSigma, Darmstadt, Germany) and analyzed by colorimetry using UV-1280 UV-VIS Spectrophotometer (Shimadzu Scientific Instruments, Pleasanton, CA).

2.9. Dual isotopes (^{15}N and ^{18}O) of NO_3^-. Both natural stable isotopes ^{15}N and ^{18}O have been used as complementary tools to investigate soil denitrification for their precise determinations, while allowing for measurements of the relative contributions of man-made and natural NO_3^- sources. It has been successfully used in agricultural systems for studying managed aquifer recharge.[29] In our study site, porewater (>15ml) was taken using 50 ml centrifuge tube at four soil depth (10; 20; 60; 100 cm) before, 24h and 48h after flooding in the fields in March 2021, and then filtered using a 0.2μm Whatman filter (MilliporeSigma, Darmstadt, Germany). Before further processing, the NO_2^- was first removed from all filtered samples using sulfamic acid (99%, MilliporeSigma, Darmstadt, Germany) with a ratio of 40:1 (40 ul of 4 mM sulfamic acid for 1ml sample with ~10 mM NO_2^-) and incubated for 20 min at room temperature.[30] Samples were neutralized using 0.5 M NaOH solution to a pH 6.5-7.5 and frozen at -20 °C until isotopic analyses. Isotope ratios of ^{15}N and ^{18}O of these pre-treated samples were measured using the bacterial method[31, 32] at UC Davis Stable Isotope Facility using the Thermo Scientific Delta V Plus isotope-ratio mass spectrometer (Bremen, Germany). The calibration standards are the nitrates USGS 32, USGS 34,

and USGS 35, supplied by National Institute of Standards and Technology, Gaithersburg, MD, USA. The enrichment factors calculation was reported in previous agricultural managed aquifer recharge study.[29]

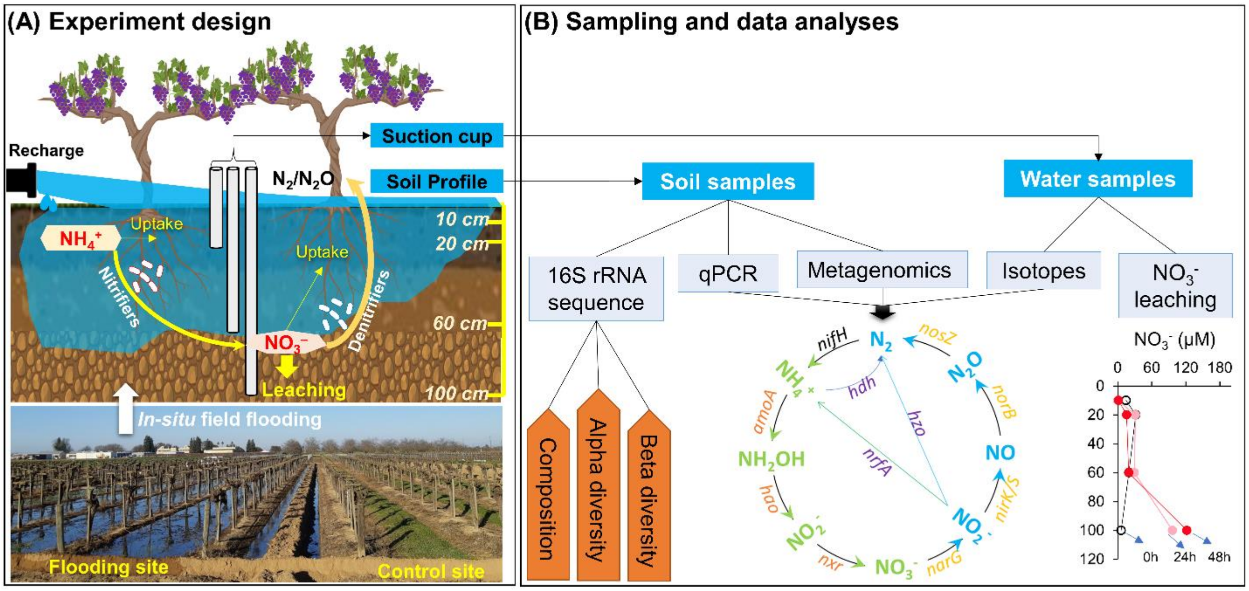

Figure. S1. Schematic flowchart shows (A) the experiment design, (B) soil/water sampling in the field and data analyses. *In-situ* field flooding was conducted for 2/4 weeks in two vineyards, California. Natural dual isotopes of water NO_3^- ($^{15}N/^{14}N$ and $^{18}O/^{16}O$) with soil 16S rRNA sequencing, metagenomics, and quantitative PCR analyses were employed to investigate the activities, taxon profiles and environmental controls of soil microbiome associated with NO_3^- leaching at four soil depths (10, 20, 60, 100 cm).

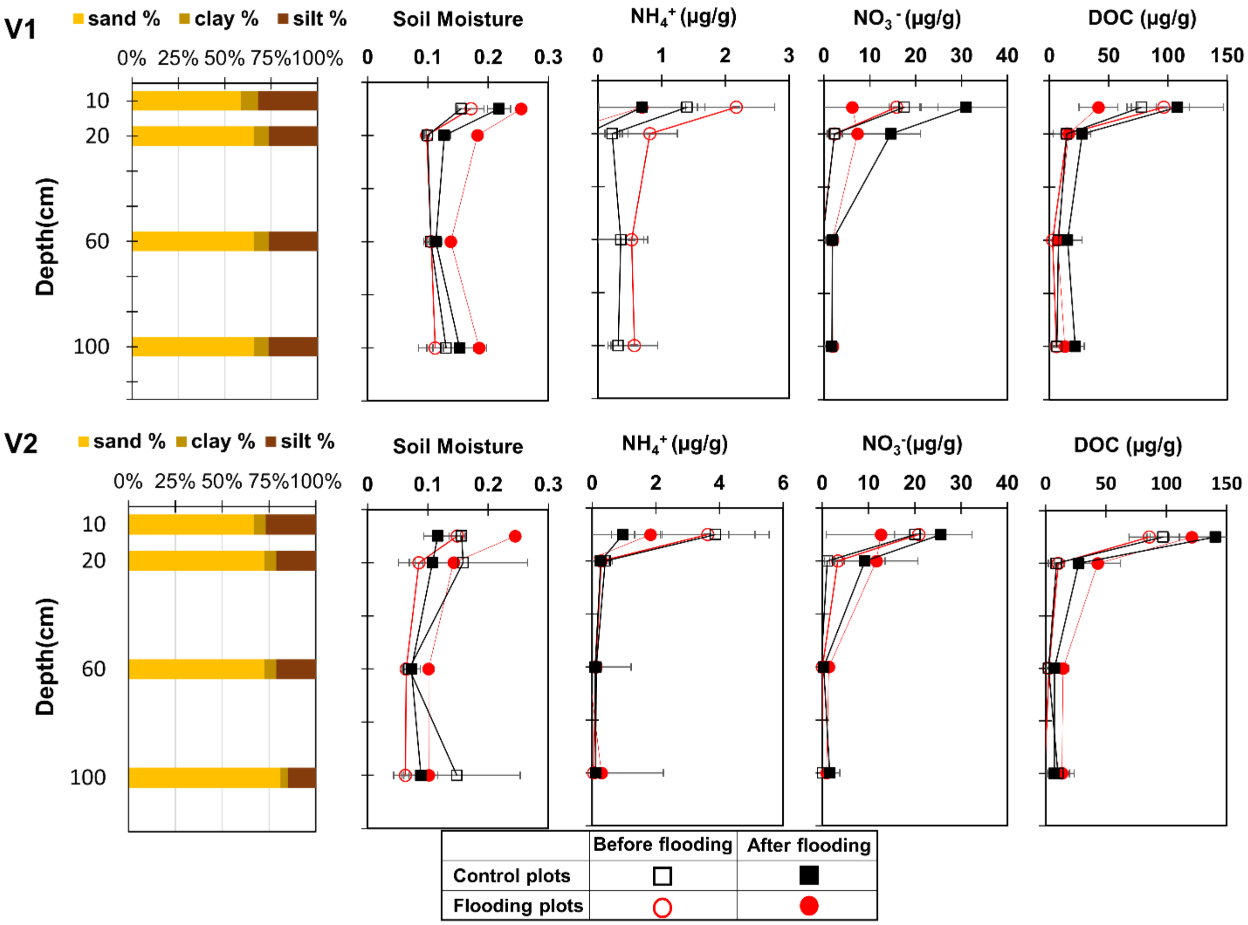

Figure. S2. The soil texture, and the changes in soil moisture, the concentrations of NH_4^+, NO_3^- and DOC at four soil depths (10, 20, 60, 100 cm) before and after flooding in two vineyards. V1, large vineyard; V2, small vineyard. Control plots had 6 replicates, and flooding plots had 12 replicates at each depth.

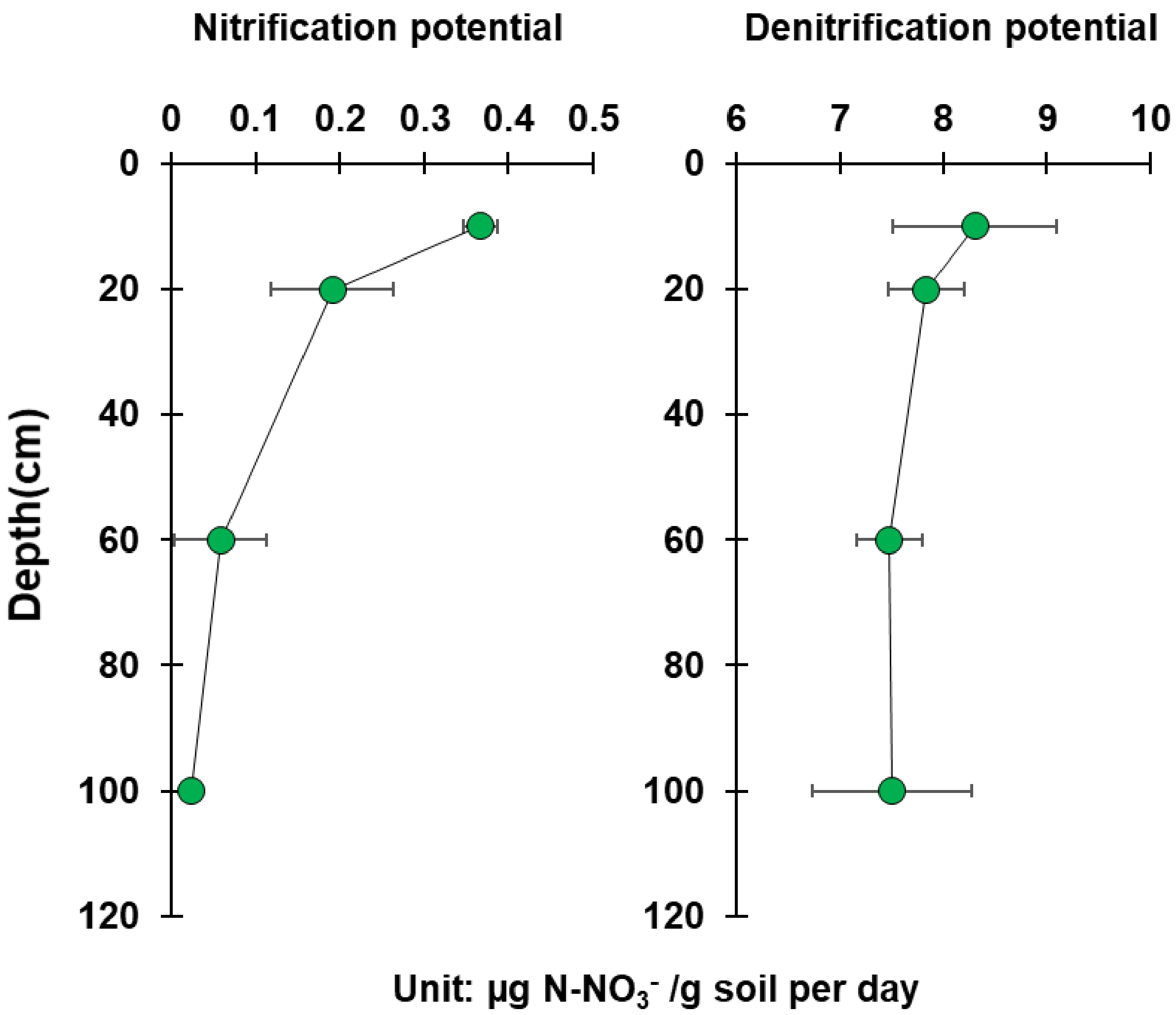

Figure. S3. The nitrification and denitrification potential measured in lab soil incubation at four soil depths (10, 20, 60, 100 cm) in V1 (large vineyard).

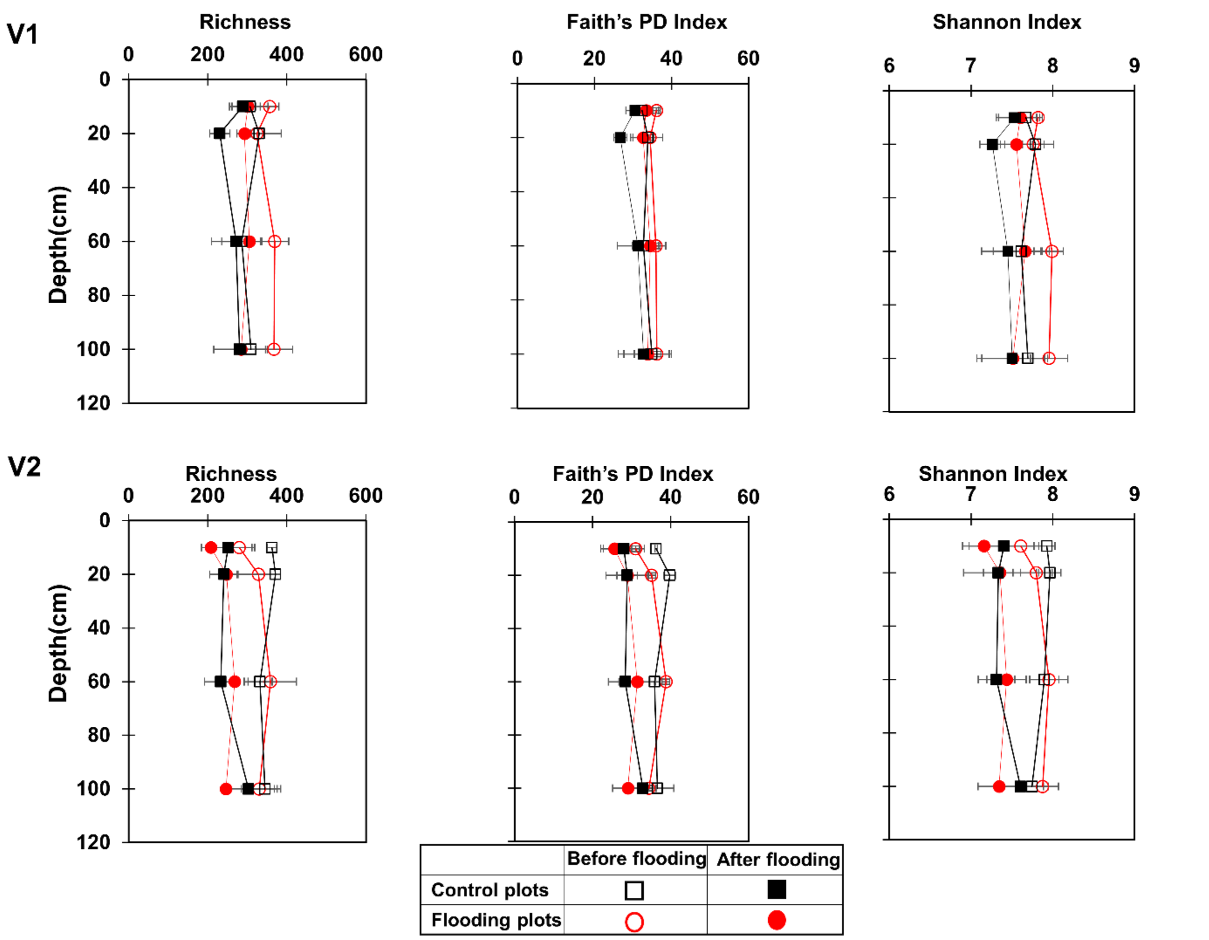

Figure. S4. Changes in the α-diversities (Richness [unit: reads]; Faith's PD Index; Shannon Index) at four soil depths (10, 20, 60, 100 cm) before and after flooding in two vineyards. V1, large vineyard; V2, small vineyard. Control plots had 6 replicates, and flooding plots had 12 replicates at each depth.

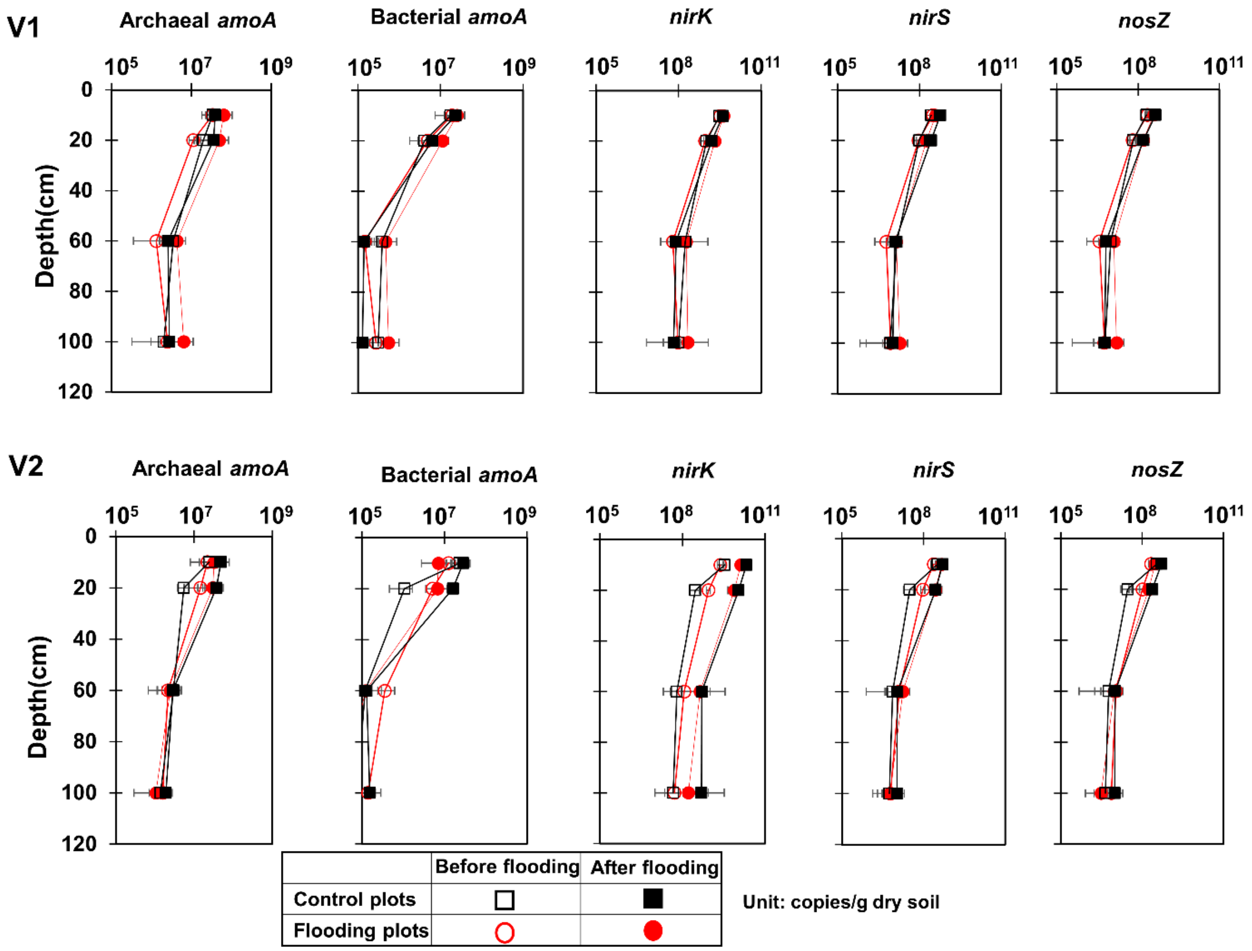

Figure. S5. Changes in functional gene abundance related to nitrification (bacterial and archaeal *amoA*) and denitrification (*nirk*, *nirS*, *nosZ*) at four soil depths (10, 20, 60, 100 cm) before and after flooding in two vineyards. V1, large vineyard; V2, small vineyard. Control plots had 6 replicates, and flooding plots had 12 replicates at each depth.

Table S1 The summary of the Permanova test of the β-diversity (Bray Curtis distances) for different flooding events

Vineyard	Depth	Flooding events	Df	replicates	Sums Of Sqs[a]	F.Model	R2	p.value	p.adjusted	sig
V1	10 cm	before vs after flooding	1	12	0.225	1.163	0.077	0.200	1.000	No
	20 cm	before vs after flooding	1	12	0.226	0.950	0.064	0.497	1.000	No
	60 cm	before vs after flooding	1	12	0.348	1.135	0.075	0.205	1.000	No
	100 cm	before vs after flooding	1	12	0.409	1.234	0.087	0.095	1.000	No
V2	10 cm	before vs after flooding	1	12	0.273	1.294	0.085	0.103	1.000	No
	20 cm	before vs after flooding	1	12	0.299	1.550	0.114	0.028	1.000	No
	60 cm	before vs after flooding	1	12	0.338	1.054	0.070	0.330	1.000	No
	100 cm	before vs after flooding	1	12	0.290	1.109	0.073	0.263	1.000	No

a, Squared distance between each paired replicate in the two compared groups; V1, large vineyard; V2, small vineyard.

Table S2 The summary of the Permanova test of the β-diversity (Bray Curtis distances) for different depths (cm)

Vineyard	Depth pairs	Df	replicates	Sums Of Sqs[a]	F.Model	R2	p.value	p.adjusted	sig
V1	10 vs 20	1	12	2.737	13.371	0.218	0.000	0.001	**
	10 vs 100	1	12	3.525	13.581	0.228	0.000	0.001	**
	10 vs 60	1	12	4.440	17.532	0.268	0.000	0.001	**
	20 vs 100	1	12	2.723	9.879	0.177	0.000	0.001	**
	20 vs 60	1	12	2.944	10.960	0.186	0.000	0.001	**
	100 vs 60	1	12	0.853	2.613	0.054	0.000	0.001	**
V2	10 vs 20	1	12	2.726	12.867	0.219	0.000	0.001	**
	10 vs 100	1	12	4.845	18.608	0.279	0.000	0.001	**
	10 vs 60	1	12	5.371	22.086	0.315	0.000	0.001	**
	20 vs 100	1	12	2.974	11.081	0.194	0.000	0.001	**
	20 vs 60	1	12	4.211	16.815	0.268	0.000	0.001	**
	100 vs 60	1	12	1.268	4.264	0.082	0.000	0.001	**

a, Squared distance between each paired replicate in the two compared groups; V1, large vineyard; V2, small vineyard.

Reference

(1) Verdouw, H.; Van Echteld, C.J.A.; Dekkers, E.M.J. Ammonium determination based on indophenol formation with sodium salicylate. *Water Res.* **1978**, 12, 399–402.
(2) García-Robledo, E.; Corzo, A.; Papaspyrou, S. A fast and direct spectrophotometric method for the sequential determination of nitrate and nitrite at low concentrations in small volumes. *Mar. Chem.* **2014**, 16230–36.
(3) Parada, A.E.; Needham, D.M.; Fuhrman, J.A. Every base matters: assessing small subunit rRNA primers for marine microbiomes with mock communities time series and global field samples. *Environ. Microbiol.* **2016**, 18(5), 1403–1414.
(4) Apprill, A.; McNally, S.; Parsons, R.; Weber, L. Minor revision to V4 region SSU rRNA 806R gene primer greatly increases detection of SAR11 bacterioplankton. *Aquat. Microb. Ecol.* **2015**, 75(2), 129–137.
(5) Caporaso, J.G.; Lauber, C.L.; Walters, W.A.; Berg-Lyons, D.; Lozupone, C.A.; Turnbaugh, P.J.; Fierer, N.; Knight, R. Global patterns of 16S rRNA diversity at a depth of millions of sequences per sample. *Proc. Natl. Acad. Sci. USA.* **2011**, 108 (Supplement 1), 4516–4522.
(6) Bolyen, E.; Rideout, J.R.; Dillon, M.R.; Bokulich, N.A.; Abnet, C.C.; Al-Ghalith, G.A.; Alexander, H.; Alm, E.J.; Arumugam, M.; Asnicar, F.; Bai, Y. Reproducible interactive scalable and extensible microbiome data science using QIIME 2. *Nat. Biotechnol.* **2019**, 37, 852–857.
(7) Callahan, B.J.; McMurdie, P.J.; Rosen, M.J.; Han, A.W.; Johnson, A.J.A.; Holmes, S.P. DADA2: high–resolution sample inference from Illumina amplicon data. *Nat. Methods* **2016**,13(7), 581–583.
(8) Katoh, K.; Standley, D.M. MAFFT multiple sequence alignment software version 7: improvements in performance and usability. *Mol. Biol. Evol.* **2013**, 30(4), 772–780.
(9) Price, M.N.; Dehal, P.S.; Arkin, A.P. FastTree 2–approximately maximum–likelihood trees for large alignments. *PLoS One* **2010**, 5(3), e9490.
(10) Quast, C.; Pruesse, E.; Yilmaz, P.; Gerken, J.; Schweer, T.; Yarza, P.; Peplies, J.; Glöckner, F.O. The SILVA ribosomal RNA gene database project: improved data processing and web–based tools. *Nucleic Acids Res.* **2013**, 41(D1), D590–D596.
(11) McMurdie, P.J.; Holmes, S. phyloseq: an R package for reproducible interactive analysis and graphics of microbiome census data. *PLoS One* **2013**, 8(4), e61217.
(12) Oksanen, J.; Blanchet, F.G.; Kindt, R.; Legendre, P.; Minchin, P.R.; O'hara, R.B.; Simpson, G.L.; Solymos, P.; Stevens, M.H.H.; Wagner, H. Vegan: Community Ecology Package, R package version 2.0-10. **2013**, 2 (9), 1–295.
(13) Martinez Arbizu, P. PairwiseAdonis: Pairwise multilevel comparison using adonis. R package version 04. **2020**.
(14) Chen, S.; Zhou, Y.; Chen, Y.; Gu, J. fastp: an ultra–fast all–in–one FASTQ preprocessor. *Bioinformatics.* **2018b**, 34(17), i884–i890.
(15) Wood, D.E.; Lu, J.; Langmead, B. Improved metagenomic analysis with Kraken 2. *Genome Biol.* **2019**, 20(1), 1–13.
(16) Lee, M. bit: a multipurpose collection of bioinformatics tools. *F1000Research* **2022**, 11(122), 122.
(17) Xue, C.X.; Lin, H.; Zhu, X.Y.; Liu, J.; Zhang, Y.; Rowley, G.; Todd, J.D.; Li, M. and Zhang, X.H. DiTing: a pipeline to infer and compare biogeochemical pathways from metagenomic and metatranscriptomic data. *Front. microbiol.* **2021**, 12, 698286.

(18) Langmead, B.; Salzberg, S.L. Fast gapped–read alignment with Bowtie 2. *Nat. Methods.* **2012**, 9(4), 357–359.
(19) Chamberlain, S.A.; Szöcs, E. taxize: taxonomic search and retrieval in R. *F1000Research* **2013**, 2.
(20) Orellana, L.H.; Chee-Sanford, J.C.; Sanford, R.A.; Löffler, F.E.; Konstantinidis, K.T. Year–round shotgun metagenomes reveal stable microbial communities in agricultural soils and novel ammonia oxidizers responding to fertilization. *Appl. Environ. Microbiol.* **2018**, 84(2), e01646–17.
(21) Yu, G.; Smith, D.K.; Zhu, H.; Guan, Y.; Lam, T.T.Y. ggtree: an R package for visualization and annotation of phylogenetic trees with their covariates and other associated data. *Methods Ecol. Evol.* **2017**, 8(1), 28–36.
(22) Baker, M.E.; King, R.S. A new method for detecting and interpreting biodiversity and ecological community thresholds *Methods Ecol. Evol.* **2010**, 1(1) 25–37.
(23) Rotthauwe, J.H.; Witzel, K.P.; Liesack ,W. The ammonia monooxygenase structural gene amoA as a functional marker: molecular fine–scale analysis of natural ammonia–oxidizing populations. *Appl. Environ. Microbiol.* **1997**, 63, 4704–4712.
(24) Tourna, M.; Freitag, T.E.; Nicol, G.W.; Prosser, J.L. Growth activity and temperature response of ammonia–oxidizing archaea and bacteria in soil microcosms. *Environ. Microbiol.* **2008**, 10, 1357–1364.
(25) Throbäck, I.N.; Enwall, K.; Jarvis, A.; Hallin, S. Reassessing PCR primers targeting nirS nirK and nos Z genes for community surveys of denitrifying bacteria with DGGE. *FEMS Microbiol. Ecol.* **2004**, 49, 401–417.
(26) Huang, L.; Bae, H.S.; Young, C.; Pain, A.J.; Martin, J.B.; Ogram, A. Campylobacterota dominate the microbial communities in a tropical karst subterranean estuary with implications for cycling and export of nitrogen to coastal waters. *Environ. Microbiol.* **2021b**, 23(11), 6749–6763.
(27) Verchot, L.V.; Holmes, Z.; Mulon, L.; Groffman, P.M.; Lovett, G.M. Gross vs net rates of N mineralization and nitrification as indicators of functional differences between forest types. *Soil Biol. Biochem.* **2001**, 33(14), 1889–1901.
(28) Petersen, D.G.; Blazewicz, S.J.; Firestone, M.; Herman, D.J.; Turetsky, M.; Waldrop, M. Abundance of microbial genes associated with nitrogen cycling as indices of biogeochemical process rates across a vegetation gradient in Alaska. *Environ. Microbiol.* **2012**, 14(4), 993–1008.
(29) Gorski, G.; Fisher, A.T.; Beganskas, S.; Weir, W.B.; Redford, K.; Schmidt, C.; Saltikov, C. Field and laboratory studies linking hydrologic geochemical and microbiological processes and enhanced denitrification during infiltration for managed recharge. *Environ. Sci. Technol.* **2019**, 53(16), 9491–9501.
(30) Granger, J.; Sigman, D.M. Removal of nitrite with sulfamic acid for nitrate N and O isotope analysis with the denitrifier method Rapid Communications in Mass Spectrometry: An International Journal Devoted to the Rapid Dissemination of Up-to-the-Minute. *Res. Mass Spectrom.* **2009**, 23(23), 3753–3762.
(31) Sigman, D.M.; Casciotti, K.L.; Andreani, M.; Barford, C.; Galanter, M.B.J.K.; Böhlke, J.K. A bacterial method for the nitrogen isotopic analysis of nitrate in seawater and freshwater. *Anal. Chem.* **2001**, 73(17), 4145–4153.
(32) Casciotti, K. L.; Sigman, D. M.; Hastings, M. G.; Böhlke, J. K.; & Hilkert, A. Measurement of the oxygen isotopic composition of nitrate in seawater and freshwater using the denitrifier method. *Analyt. chemist.* **2002**, 74(19), 4905-4912.
